# Protein Expression Analysis in Reversible Photobleached Cells of *Scenedesmus vacuolatus* after High Temperature Stress

**DOI:** 10.3390/ijms20123082

**Published:** 2019-06-24

**Authors:** Tzu-Hsing Ko, Kuen-Lin Leu, Ban-Dar Hsu, Tzan-Chain Lee

**Affiliations:** 1 Anxi College of Tea Sciences, Fujian Agriculture and Forestry University, Fuzhou 350002, China; hsingko@gmail.com; 2 Department of Cosmetic Science, Chia Nan University of Pharmacy and Science, No. 60, Section 1, Erren Rd. Rende Dist., Tainan City 71710, Taiwan; kuenlin@mail.cnu.edu.tw; 3 Department of Life Science, National Tsing Hua University, No.101, Section 2, Kuang-Fu Road, Hsinchu City 30013, Taiwan; lshbd@life.nthu.edu.tw

**Keywords:** ATP synthase, chlorophyll, heat shock protein (HSP), heat stress, photobleaching, protein identification

## Abstract

We have analyzed protein expression in the bleached small vegetative cells of synchronous *Scenedesmus vacuolatus* to investigate how unicellular algae lived through stress. These cells were subjected to heat treatment (46.5 °C for 1h in dark condition) and then cultured under continuous illumination for 24 h. Flow cytometry analysis of the chlorophyll autofluorescence intensity of *S. vacuolatus* cells indicated that heat-treated cells were completely bleached within 24 h of light cultivation. Transmission electron microscopy (TEM) images showed that bleached cells maintained thylakoid membrane structure, but with lower contrast. The bleached cells regained green color after 72 h, along with a recovery in contrast, which indicated a return of photosynthetic ability. Two-dimensional gel electrophoresis (2DE) showed that the protein expression patterns were very difference between control and bleached cells. ATP synthase subunits and glutamine synthetase were down-regulated among the many differences, while some of phototransduction, stress response proteins were up-regulated in bleached cells, elucidating bleached cells can undergo changes in their biochemical activity, and activate some stress response proteins to survive the heat stress and then revive. In addition, small heat shock proteins (HSPs), but not HSP40 and HSP70 family proteins, protected the bleaching cells.

## 1. Introduction

Green algae are growing in a variety of habitats and are widely distributed around the earth. They can survive under many stress conditions and reestablish new populations when conditions are favorable. For instance, desiccated desert green algae can survive four weeks in darkness, and then recover photosynthetic activity within 1 h of rehydration [1]. Similarly, terrestrial algae, *Prasiola calophylla*, lost the quantum yield in desiccation stress and it can completely recover within 100 min. after rehydration [2]. Soil alga *Rhizoclonium crassipellitum* was found to be absent from summer to winter, and it appeared in spring of next year [3]. Aquatic algae Chlorella can degenerate chloroplasts to accommodate the changes in environment and regenerate the organelle under favorable condition [4].

Heat stress is a general stress in natural environment and cultivation systems (e.g., photobioreactors, open ponds). Torzillo et al. [5] reported that the temperature of an outdoor photobioreactor could be higher than 40 °C for several hours per day in the summer in Firenze (Italy). Tredici and Materassi [6] also observed a temperature as high as 56 °C in the vertical alveolar panels.

In chloroplasts, heat stress affects the metabolism of reactive oxygen species (ROS) and inactivates photosystem II (PSII) and Rubisco activase [7,8,9]. ROS are signal molecules. At low concentration, they can activate stress protection mechanisms in cells [10,11]. However, large amounts of ROS can attack cell membrane systems, proteins and nucleic acids induce cell damage. Plants and algae can activate many stress response genes (heat stress transcription factors) and synthesize protective proteins (e.g., heat shock proteins, superoxide dismutase) to stabilize protein structure and remove harmful substrate in order to cope with heat stress [12,13]. 

In a previous report, the synchronous small cells of *S. vacuolatus* were subjected to a heat treatment (46.5 °C for 1 h in the dark) and then cultured right away under continuous illumination [14]. It was found that the heat treatment inhibited the photosynthetic activity and the illumination during cultivation at an intensity that was normally not harmful, induced chlorophyll degradation [14]. Nevertheless, the *S. vacuolatus* cells, after going through the heat stress and continuous light cultivation, were able to maintain their cell membrane integrity and become green again after 72 h [14]. In this study, the changes of chlorophyll fluorescence intensity of the cell population, the structure of thylakoid membranes, and protein compositions before stress (control) and after stress (bleached) were investigated. The results suggest that these bleached *S. vacuolatus* cells down-regulated their biochemical activity, and they displayed a different protein expression pattern. This transformation may help the bleached cells to revive under suitable conditions.

## 2. Results

### 2.1. Population Responses.

**Figure 1:**
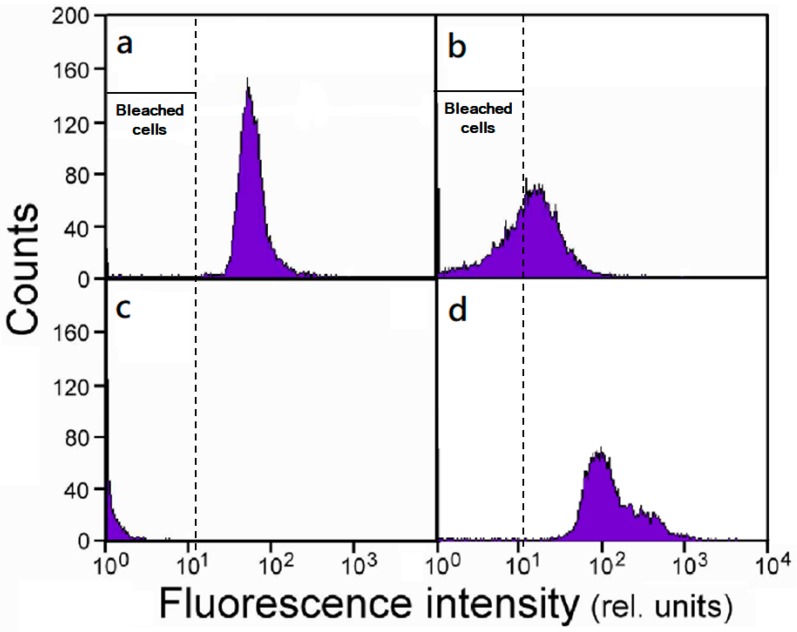
The frequency distribution histograms of *Scenedesmus* cells analyzed for chlorophyll *a* fluorescence. (**a**) Untreated synchronous small cells, and the cells that had been heat treated (46.5 °C, 1 h) and then cultured under illumination for (**b**) 6 h, (**c**) 24 h, and (**d**) 96 h. A fluorescence intensity lower than 20 are identified as bleached cells. Three independent measurements were conducted at each time point.

*S. vacuolatus* cells that were subjected to a heat treatment were immediately cultured under continuous illumination, and the flow cytometry analyzed for chlorophyll a fluorescence was used to continuously monitor the changes in the chlorophyll content of the cell population. Here, we took the fluorescence intensity of 20 (relative units) as the dividing line to distinguish between the green and bleached cells. Figure 1 depicts the frequency distribution of the fluorescence intensity of control cells and the heat treated cells that had been cultured for various times. After light culture for 6 h, the shift of distribution toward the left indicated cells were losing chlorophylls (Figure 1b). Until 24 h, all of the heat treated cells were bleached (Figure 1c). However, starting from 24 h of cultivation, an increase in the high-fluorescent cell population becomes progressively significant (Figure 1d). Note that a fraction of these newly emerged cells could emit fluorescence at an intensity (~300 in relative unit) that was about five times higher than the control (~60). This might have something to do with the new cells population was not synchronous, and continued growth could generate cells that contain multiple daughter cells/chloroplasts.

### 2.2. Morphological Studies of Thylakoid Membranes. 

**Figure 2:**
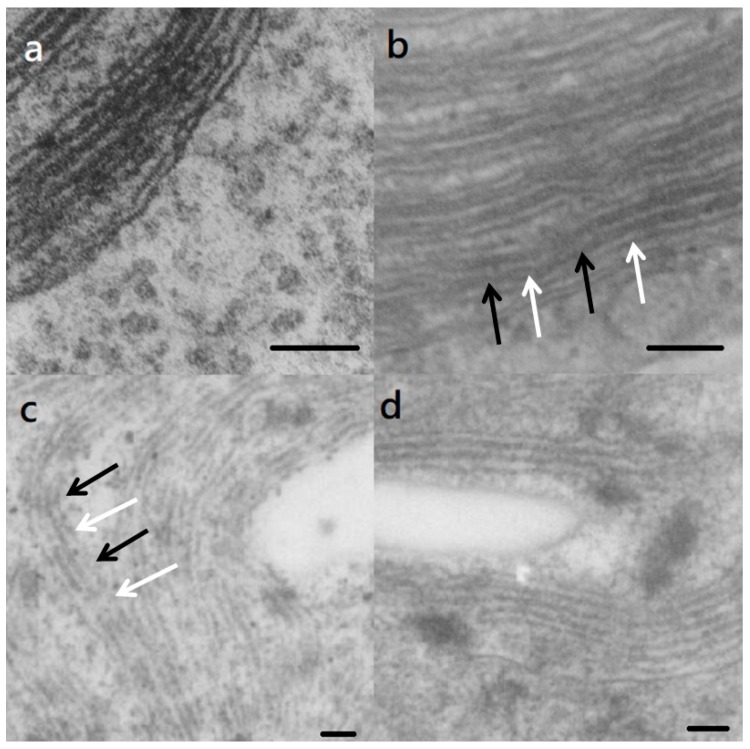
Transmission electron micrographs of the thylakoids of (**a**) freshly harvested *S. vacuolatus* (control) cell and the cells that had been heat-treated and then cultured under illumination for (**b**) 6 h, (**c**) 24 h, and (**d**) 72 h. Bars = 100 nm. The light and dark segments are indicated by white and black arrows, respectively.

The TEM study found that the *S. vacuolatus* cell has typical triplet parallel thylakoid membranes in chloroplast (Figure 2a). After light cultivating for 6 h, the light and dark segments showed up along the length of thylakoid membranes (Figure 2b,c, arrows). In bleached cell (light cultivated for 24 h), thylakoid membranes appeared to maintain intact, but with lower contrast when compared with the control image (Figure 2c). At a longer time of cultivation, the thylakoid membranes in the recovered cells showed a partial recovery, in contrast (Figure 2d).

### 2.3. Proteins Expression Analysis. 

**Figure 3:**
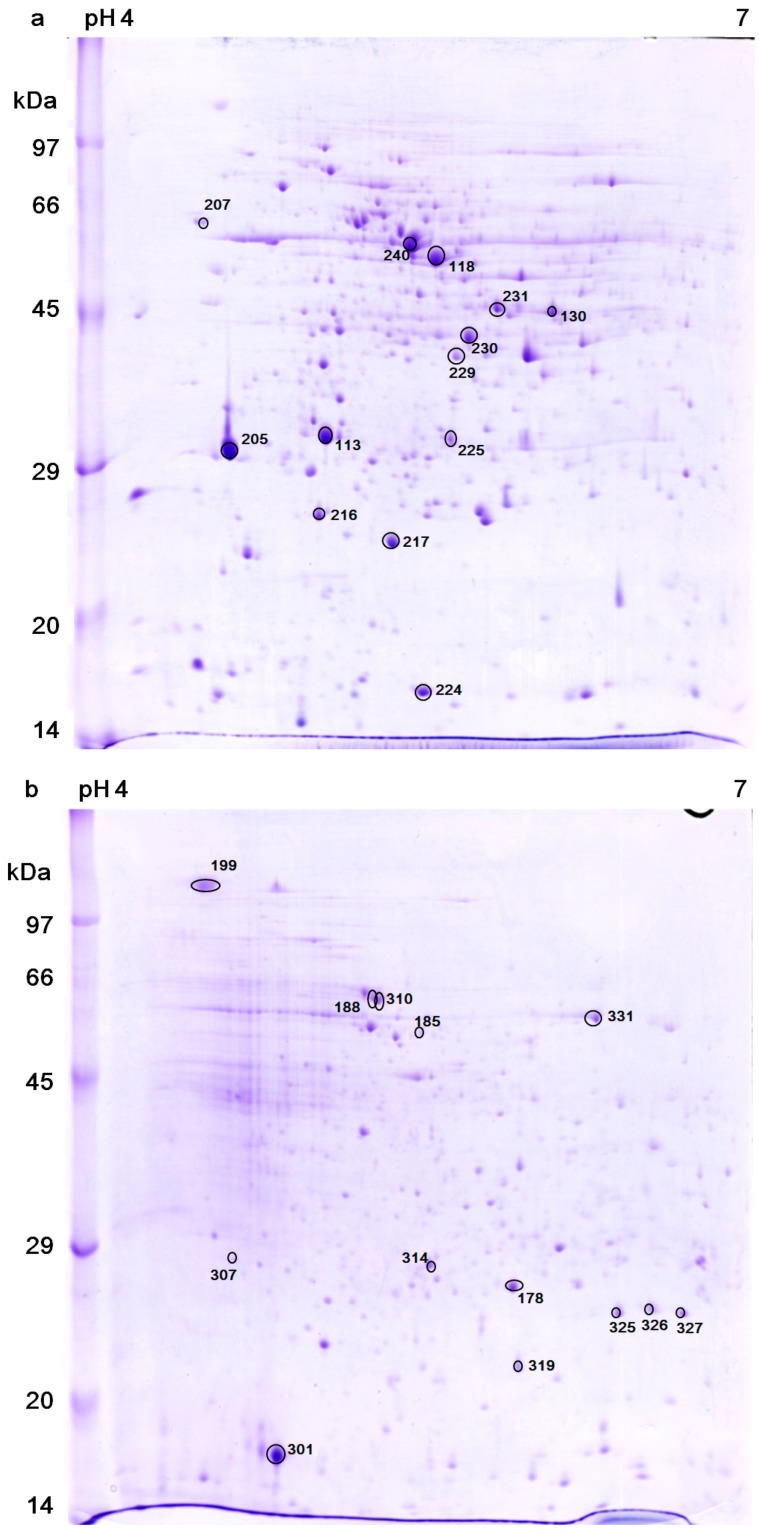
Two dimensional gel electrophoresis (2DE) analysis of the total protein extracts. (**a**) Freshly harvested (control) cells and (**b**) the bleached cells that had been heat-treated (46.5 °C for 1 h) and then cultured under illumination for 24 h. Identified protein spots were marked with numbers.

**Figure 4:**
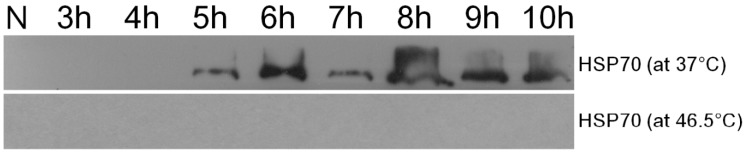
Western blotting analysis of the HSP70 in heat-treated *Scenedesmus* cells. Cells (1 × 10^7^) were either subjected to 37 °C treatment for various times (upper panel) or 46.5 °C for one hour then cultivated at 32 °C for various time (lower panel). N, normal cell.

Figure 3a,b showed the two dimensional gel electrophoresis (2DE) of the control cells and the bleached cells, respectively. The patterns of spots are quite different between the two samples; this might be a result of protein modification, new expression and/or down regulation. 150 highly expressed proteins were selected for protein identification, but only 26 of them were identified. The protein spots expression levels that were identified by using LC-MS/MS and MALDI-TOF MS are listed and annotated in Table 1 and Table 2, respectively. Protein analysis reveals that bleached cells down-regulated several important metabolism-related proteins, such as ATP synthase β, α, b subunits (protein No. 118, 240, 216, respectively), glutamine synthetase (No. 130), tryptophanyl- tRNA synthetase (No. 230), and dnaJ (heat shock protein 40 family) homolog protein (No. 229), which suggested that these cells actually entered a low activity state upon bleaching. On the other hand, the bleached cells mainly up-regulated the transcription factors or stress response proteins, including a most abundant protein “water stress-inducible protein Rab21” (No. 301), stress response proteins (No. 310, 326), light response transcription factors (e.g., No. 307, 314), and small HSP (e.g., No. 326), which indicated that these cells had entered a protective state against a harsh environment. In addition, western blot analysis found that HSP70 can be induced when the cells were cultured at a slightly higher temperature (37 °C instead of 32 °C) for 5 h. However, HSP70 could not be detected in the cells that had been heat treated at 46.5 °C for 1 h, followed by continuous light culture at 32 °C (Figure 4).

## 3. Discussion

The heat treatment completely inhibits the light reactions of photosynthesis and temporarily represses cell multiplication [14]. Previous reports suggest that the targets of thermal damage are at PSII with its oxygen-evolving complex, ATP generating system, and carbon fixation by Rubisco [7,15].

The subsequent light cultivation induced the complete chlorophyll degradation in all cells within 24 h (Figure 1a–c). The process was only found in light cultivation, but not in dark cultivation [16], which indicated that chlorophyll degradation proceeded through photooxidation [17]. However, flow cytometry analysis found some of newly emerged cells in 85 h culture that could emit chlorophyll fluorescence at an intensity that was much higher than the control (Figure 1d). This can be explained by the observation in one of our previous TEM study, which found that, among the cell population from 72 h culture, there were many cells that contained several chloroplasts in the regreening cells [14]. 

Chlorophylls can turn into harmful pigments if algae cells are under irradiation when photosyhthetic ability is completely inhibited. This is because chlorophyll can be photooxidized in the absence of electron transport. The electrons would be transferred to oxygen, which leads to the formation of ROS [18,19]. These ROS are primarily generated in chloroplasts and can cause oxidative damage. Therefore, thylakoid membranes and the proteins within them are the targets of damage that is caused by exceed light [20]. The loss of chlorophylls was accompanied by structural changes in chloroplasts. In this study, the thylakoid membranes of the bleached cells still maintained their integrity, lost contrast with the background, and showed black-white bands along their length (Figure 2b,c). Notably, these black-white bands only presented on thylakoid membrane, but not in lumen. Similar phenomena were observed when the plants are under stress [21,22,23]. After regreening, the appearance of the thylakoid membranes of heat-treated cells was largely restored to that of control cells (Figure 2d). It is worth mentioning that photooxidation can also regulate the gene expression of some pigments’ biosynthetic pathway [24]. In this study, we found dihydroflavonol 4-reductase (protein No. 178), which is a protein that regulates flavonoid metabolism were up-regulated in bleached cells.

The proteins profile was another significant change brought about by heat plus light stress (Figure 3a,b). In 2DE, many of the proteins decreased in amount or disappeared completely, and many new spots were synthesized in the bleached cells (Table 2), which indicated that these bleached cells were functionally active and had entered a different physiological state. 

F_O_F_1_ ATP synthase is located in mitochondria inner membranes and chloroplast thylakoids. The F_1_ subunit α (No. 240), *β* (No. 118) and F_O_ subunit b (No. 216) of ATP synthase were down-regulated in the bleached cells (Table 1 and Table 2). This is in line with our previous finding that mitochondria were only partially inhibited, but photosynthesis had been completely inhibited, so the source of ATP could only come from the mitochondria in bleached cells [14]. The presence of partially operating mitochondria implied that these bleached cells were still functioning to a certain extent, such as generating the energy that is necessary for maintaining integrity of cell membranes. 

Tryptophanyl-tRNA synthetase (No. 230) can catalyze the aminoacylation of tRNA, which is an enzyme that is associated with protein synthesis [25]. The enzyme was not detected in the bleached cells (Table 2). It might be that the protein expression level became too low to be detected by Coomassie Brilliant Blue, or a protein modification changed its location in the 2DE gel. In addition, the expression of glutamine synthetase (No. 130) was also down-regulated. These results indicated that these bleached cells have down-regulated their bioactivity and protein expression. A similar result can be observed in another unicellular algae *Chlamydomonas reinhardtii* after heat stress [15].

Maximal photosynthetic activity (Fv/Fm) and chlorophyll contents were gradually recovered after light culture for 24 h [14]. It is likely that PSII and light-harvesting complex (LHC) were synthesized in bleached cells in this time period, which required newly synthesized chlorophylls to bind with the chlorophyll a–b binding proteins of LHC. ALBINO3-like protein 1 (No. 188) is necessitated for the insertion of LHC proteins into the thylakoid membrane and assembly of LHC I and II [26]. The increasing of this protein expression level might indicate that the assembly of LHCs is enhanced in the bleached cells.

HSPs can stabilize protein structure and their expression levels are changed under abiotic stresses. In this study, a member of HSP40 protein family, dnaJ protein P58IPK homolog protein (No. 229), was down-regulated, while chloroplast HSP26.2 (No. 326) was up-regulated in the bleached cells (Table 2). Moreover, HSP70 could not be detected in the cells that had been heat treated at 46.5 °C for 1 h, followed by continuous light culture at 32 °C (Figure 4). Similar results were observed in unicellular algae, *C. reinhardtii*, in which many HSP60, HSP70, and HSP100 family proteins were down-regulated under irradiance stress [27]. The results indicated that the dnaJ protein (No. 229) and HSP70 might not function as protective proteins in the bleached cells. However, chloroplast HSP26 was inducible by heat stress and it has been found to interact with ATP synthase α, β subunits, chlorophyll a–b binding proteins of LHC, oxygen-evolving enhancer protein 1 of PSII, and PSI subunit VI protein [28,29]. As mentioned above, ATP could only come from mitochondria in the bleached cells. This information might imply that the ATP synthase in chloroplast maintains a “unwork state” in bleached cells. The overexpression of HSP26 in transgenic tobacco could alleviate photoinhibition under extreme temperatures [30], and protected PSII activity (Fv/Fm) during heat and oxidative stress in tall fescue [31]. In the bleached cells, chloroplast HSP26.2 (No. 326) might play a role in protecting chloroplast and cell regreening.

A reversible shrinkage of the cytoplasm was observed between 2 and 24 h of light cultivation in the heat treated cells [14]. This phenomenon is generally observed in osmotic stressed or water stressed cells. In this study, the most abundant protein in bleached cells is water stress-inducible protein Rab21 (Responsive to abscisic acid 21; No. 301; Table 2). The protein was only located in cytosol and can be induced by water stress [32]. Many reports showed that abscisic acid (ABA) could improve the tolerance of unicellular algae to dehydration, osmotic stress, and oxidative stress [33,34,35,36,37], and also increase biomass production in *Chlamydomonas* and *Scenedesmus* cells [38,39]. In another thread, proton pump-interactor 1(PPI1), which is the second abundant protein in bleached cells, was also up-regulated (No. 199; Table 1). In *Solanum tuberosum* L., drought stress slightly increases the *StPPI1* mRNA expression level [40]. PPI1 can stimulate H^+^ATPase activity and generate a membrane potential that plays a role in the adaptation of plants to stress conditions [40,41]. The two proteins Rab21 and PPI1 may not be directly correlated with each other. However, their common interacting moleculars, ABA have two different mechanisms that regulate H^+^ATPase activity in guard cell and root cell under salt stress [42,43,44]. It is still unknown how ABA regulates H^+^ATPase activity in unicellular cells. We suggest that these proteins’ up-regulation might be associated with the shrinkage of the cytoplasm we observed, indicating that osmotic stress or water stress was present in bleached cells [14]. They all could increase stress tolerance in the bleached cells. 

Our findings can help to explain how algae survive and reproduce in the transition of environmental stress. The bleached cells in this study were found to down-regulate many biochemical activities and, in the meantime, activated some stress response proteins to survive the damage that is incurred by stress treatment. These changes in proteins’ expression pattern might elucidate many phenomena observed in the previous study [14]. 

## 4. Materials and Methods

### 4.1. Cell Culture and Heat Treatment. 

Unicellular *S. vacuolatus* (SAG 211-8b cells, obtained from the Algal Collection Center, University of Göttingen, Germany) were photoautotrophically cultured. Axenic cultures were conducted, as described in Lee and Hsu [14]. In briefly, the algae cells were grown at 32 °C with slow bubbling at 3.5% CO_2_ in air. Illumination was provided by daylight fluorescence tubes at an intensity of 150 μmol photon m^−2^s^−1^. Synchronous cultures were obtained by the programmed 14 h light/10 h dark regime. The culture started with a cell density of 2 × 10^6^ cells mL^−1^ and dilution (~15-16×) was made after each 24 h cycle. 

Heat treatment was applied by incubating the synchronous culture at the stage of small vegetative cell (5 μm in diameter) at 46.5 °C for 1 h in the dark. The heat-treated cells were cultured immediately thereafter at 32 °C with slow bubbling at 3.5% CO_2_ in air, and continuously illuminated at 100 μmol photon m^−2^s^−1^ [14]. 

### 4.2. Flow Cytometry Analysis. 

Flow cytometry analysis was carried out with a FACSCaliburTM flow cytometer (Becton Dickinson, California), which was equipped with a 15 mW argon-ion laser. For chlorophyll content measurement, cell samples (2 mL) were collected at different time points of cultivation and the fluorescence of intrinsic chlorophyll a was detected with an excitation at 488 nm and emission longer than 650 nm. Each measurement was analyzed for 10^4^ particles.

### 4.3. Transmission Electron Microscopy (TEM). 

The same procedure, as delineated in Lee and Hsu [14], was adopted. *S. vacuolatus* cells were collected by centrifugation at 1800× *g* for 5 min. at 4 °C and then fixed for 4 h in 2% glutaraldehyde in 66 mM potassium phosphate buffer (pH = 7.1) at 4 °C. The samples were washed three times at 15 min. intervals in 66 mM potassium phosphate buffer (pH = 7.1) at 4 °C and then post-fixed in 2% aqueous OsO_4_ in 66 mM potassium phosphate buffer (pH = 7.1) for 4 h at 4 °C. This was followed by three washes at 15 min. intervals with the same buffer. The tissues were dehydrated in a grade acetone series, infiltrated through acetone: Spurr resin mixtures, then embedded in pure Spurr resin, and polymerized at 70 °C for 15 h. Ultrathin gold sections were cut on a Reichert-Jung ultramicrotome, which was collected on formvar-coated grids, stained with saturated solution of uranyl acetate in 100% methanol, and post-stained with lead citrate. Observations were made while using a Hitach-7500 transmission electron microscope (Hitachi, Japan).

### 4.4. Two Dimensional Gel Electrophoresis (2DE). 

Lee and Hsu has described the same procedure [14]. *S. vacuolatus* cells (2 × 10^9^, control cells and heat-treated cells that had been cultured for 24 h under light) were collected by centrifugation at 1800× *g* for 10 min. and pulverized in liquid nitrogen. The samples were then homogenized by MagNA Lyser (Roche, Mannheim, Germany) at 6500 rpm for 30 s in 500 μL lysis buffer (7 M urea, 2 M thiourea, 4% (*w*/*v*) CHAPS ([(3-cholamidopropyl) dimetylammonio]-1-propanesulphonate), 0.5% (*w*/*v*) NP-40 (Nonidet P-40) and 10 mM Tris-HCl (pH = 8.3)), followed by centrifugation at 13,000× *g* for 10 min. to remove the debris. The protein concentrations were determined using two-dimensional (2D) Quant Kit, according to the manufacturer’s protocol (GE healthcare, San Francisco, CA, USA). Before analysis, the samples were stored at −80°C. 2DE was as described by Zhang and Koay (2008) [45], except that the first-dimensional gel separation was carried out with 18 cm pH 4-7 IPG strips. 900 μg protein was loaded per gel. The gels were visualized while using Coomassie Brilliant Blue R250 staining. 

### 4.5. Protein Expression Level Analysis. 

2DE image analysis was performed while using PowerLook 1120 scanner (UMAX, CA) and ImageMasterTM 2-D Platinum version 5.0 (Amersham Bioscience, NJ).

### 4.6. In-gel Digestion.

Referred to the procedure of Shevchenko et al. [46], but skipped the steps of reduction and alkylation. An Ettan automated spot picker from 2DE was used to pick the interesting spots, which were then put into 0.5 mL microtubes (Axygen; extrapure). The gel pieces were destained with 50 μL of 10 mM ammonium bicarbonate/50% acetonitrile and incubated with vortexing for 15 min. After removing the supernatant, 100 μL of 100% acetonitrile was added for gel pieces drying. Subsequently, the liquid was eliminated and 25 μL of 25 mM ammonium bicarbonate was added for 5 min., followed by a wash with 25 μL of 100% acetonitrile for 15 min. After removing all remaining liquid, 100 μL neat acetonitrile was added with vortexing for 3 min., until the gel pieces become white and shrinking. The gel pieces then were dried in a SpeedVac, and reswelled in 20 ng/μL sequencing grade modified procine trypsin (Promega sequencing grade modified trypsin (V511A)) in 10 mM ammonium bicarbonate for 1 h. Subsequently, the gel pieces were covered with 10mM ammonium bicarbonate and incubated at 37 °C overnight. Peptides were extracted by 15 μL of 50% acetonitrile with 1% trifluoroacetic acid for 15 min. twice, and then stored at −20 °C prior to LC-MS/MS or MALDI-TOF MS analysis.

### 4.7. Liquid Chromatography–Tandem Mass Spectrometry (LC-MS/MS). 

The procedure was performed based on the method that was described by Chan et al. [47]. The 5 μL tryptic digested protein samples were injected onto a reversed phase capillary column (PepMap 75 μm × 150 mm, LC Packings) while using a nanoflow high pressure liquid chromatography system (Ultimate, Dionex) that was connected on-line to an electrospray ionization Q-TOF I mass spectrometer (Waters). The flow rate was 300 nL min^−1^ and separation was performed by gradient elution from 5 to 50% solution B (80% (*v*/*v*) acetonitrile, 0.1% formic acid) for 60 min., followed by an isocratic step at 100% solution B for 10 min. Balance solution A was 0.1% formic acid. Data-dependent acquisition was used with a mass spectrometry scans set every second (m/z 350–1500), and MS/MS was performed on automatically selected peptide ions, also for 1 s (m/z 50–2000, continuum mode), while using the function switching in MassLynx version 4.0 software. Raw MS/MS data were smoothed (Savitzky Golay, two channels twice) and centroided (at 80%) and peaks lists were generated while using MassLynx software.

### 4.8. Matrix Assisted Laser Desorption Ionization–Time of Flight Mass Spectrometry (MALDI-TOF MS). 

The tryptic digested protein samples were resuspended in 5 μL of 0.1% formic acid. 0.65 μL of the samples were spotted onto an anchorchip target plate (600 μM/384 well, Bruker Daltonics) and further covered with 0.5 μL of matrix solution (1 mg/mL α-cyano-4-hydro xycinammic acid (HCCA) in 50% acetonitrile and 0.1% trifluoroacetic acid). The peptide calibration standard (Bruker Daltonics. m/z 757.39916, 1046.54180, 1296.68480, 1347.73540, 1619.82230, 1758.93261, 2096.08620, 2465.19630, 3147.47100) was also spotted onto the target plate for peptide calibration. The peptide mass fingerprints were obtained while using MALDI-TOF MS (autoflex III, Bruker Daltonics).

### 4.9. Protein Identification. 

The LC-MS/MS spectra were submitted to the MASCOT server (Matrix Science, v. 2.6.2) and compared against the protein database from NCBI (NCBInr 20180429). The taxonomy was set to Viridiplantae (green plants). The following parameters were used for database searches: peptide tolerance at ±1.2 Da and MS/MS tolerance at ±1.5 Da; peptide charge of 2+ or 3+; and, trypsin as enzyme allowing for up to two missed cleavages. The fixed modification is carbamidomethyl (C). Variable modification, like oxidation (M) and phosphorylation (ST), were also used for consideration through queries. MALDI-TOF MS analysis followed that outlined by Lai et al. [48]. The peaks in the mass range m/z 600–4000 were used to generate a peptide mass fingerprint that was matched to the theoretical trypsin digests of proteins from the NCBI database. The accepted protein identifications required that the Mascot scores be higher than the threshold value in MS/MS analysis or in MALDI-TOF MS analysis. Protein location and functional description were referred to UniProt Knowledgebase (https://www.uniprot.org/)

### 4.10. Western Blotting Analysis

The total proteins of 1 × l0^7^ *Scenedesmus* cells were subjected to SDS-PAGE and the resolved proteins were transferred to nitrocellulose membranes. The transfer buffer included 192 mM glycine, 25 mM Tris-HCl buffer (pH = 8.3), and 1% methanol. The mernbranes were incubated for 2 h at room temperature in blocking buffer [5% non-fat milk in TTBS buffer (150 mM NaCl, 20 mM Tris-HCl (pH = 7.4), and 0.1% Tween 20)], and then immunoblotted with 50 ng/mL anti-hsp70/hsc70 primary monoclonal antibody (Stressgen) in TTBS buffer containing 3% non-fat milk at room temperature for 2 h. The membranes were washed with 150 mM NaCl, 20 mM Tris-HCl (pH = 7.4), 0.1% Tween 20, and probed with secondary antibodies (1:3000), coupled to horseradish peroxidase (HRP) at room temperature for 1 h. The ECL system was used for detection.

## 5. Conclusions

**Figure 5:**
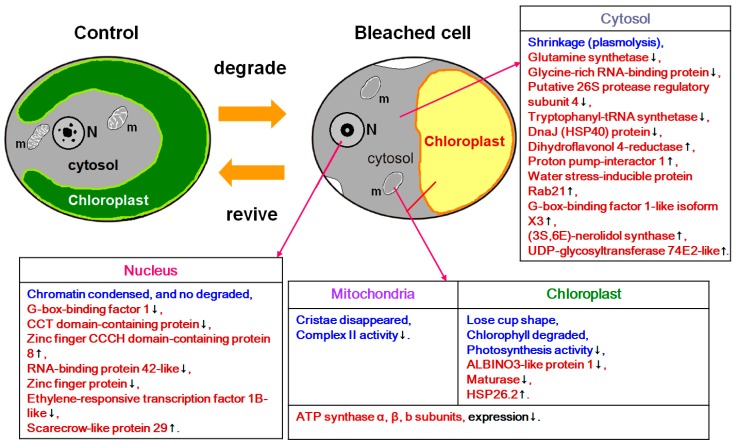
A summary of the results that combine the morphological and physiological studies from a previous report [14], and the proteins expression analysis in this study. The changes observed in the previous study are marked in blue, whereas those in the present study in red. m: mitochondria; N: nucleus.

In this study, heat treatment followed by cultivation under continuous illumination resulted in bleaching in the *S. vacuolatus* cells and the loss of photosynthetic activity. However, these bleached cell down-regulated many biochemical related proteins, such as ATP synthase α, β subunits, and glutamine synthetase, while up-regulated some phototransduction transcription factors, stress response proteins, and protective proteins, like small HSPs (but not HSP40 and HSP70 family proteins). The molecular evidence indicates that bleached cells can regulate their biochemical activity and activate certain protective mechanisms to remedy the damage that is incurred by the heat stress and successfully resurrect (Figure 5).

## Figures and Tables

**Table 1:** List of identified proteins from *S. vacuolatus* using liquid chromatography–tandem mass spectrometry (LC-MS/MS).

No.	Homologous Protein (LC/MS/MS)	Obs/Theor M. W. (kD)	Obs/Theor PI	Accession No.	Mascot Score	Identified Peptide Fragment	Location	Functional Description	Expression Fold Change (Bleached/Control)
118	ATP synthase β subunit [*Spathelia wrightii*]	55/50.118	5.52/5.14	CBY80088.1	67	K.IGLFGGAGTGK.T	Chloroplast and mitochondria	Produces ATP from ADP. The catalytic sites are primarily by the subunits.	0.02
130	Glutamine synthetase [*Chlorella sorokiniana*]	42/41.671	6.06/6.17	PRW34037.1	79	K.HETCDINTFR.F	Cytosol	Catalyzes the ATP-dependent biosynthesis of glutamine from glutamate and ammonia.	0.10
178	Dihydroflavonol 4-reductase [*Vitis davidii*]	28/38.139	5.86/5.81	AHK10250.1	66	R.ATVRDPTNVK.K	Extrinsic component of endoplasmic reticulum	This protein is involved in flavonoid metabolism.	1.13
199	Proton pump-interactor 1 [*Zea mays*]	170/170.178	4.48/4.59	ONM22629.1	60	K.AENMVEVKSAAR.E	Endoplasmic reticulum and plasma membrane	Regulation of proton transport. Enhances H^+^ATPase activity.	4.82
205	Maturase K (chloroplast) [*Trochodendron aralioides*]	28/28.035	4.57/9.98	AAB58649.1	77	R.SQMIENAFLIDSTSKKFDTIVPISPLIGSLAK.A	Chloroplast	Probably assists in splicing its own and other chloroplast group II introns. mRNA and tRNA processing.	0.04
224	Glycine-rich RNA-binding protein [*Dorcoceras hygrometricum*]	16/16.923	5.46/5.54	KZV53374.1	66	R.GGGGGGYGGDR.G	Shuttling between nucleus and cytoplasm	Plays a role in RNA transcription.	0.05
240	ATP synthase α subunit [*Eriachne mucronata*]	56/55.704	5.42/5.94	YP_009410967.1	69	K.TAIATNTILNQK.S	Chloroplast and mitochondria	Produces ATP from ADP. The α subunit is a regulator of ATP synthase.	0.01

**Table 2:** List of identified proteins from *S. vacuolatus* using matrix assisted laser desorption ionization–time of flight mass spectrometry (MALDI-TOF MS).

No.	Homologous Protein (MALDI-TOF)	Obs/Theor M. W. (kD)	Obs/Theor PI	Accession No.	Mascot Score	No. Match Peptides (Seq. Coverage)	Location	Functional Description	Expression Fold Change (Bleached/Control)
113	RNA-binding protein 42-like [*Erythranthe guttata*]	32/27.233	5.0/9.73	XP_012840025.1	83	29(49%)	Nucleus	Essential polysome-associated RNA-binding protein.	0.03
185	Zinc finger protein [*Macleaya cordata*]	55/55.293	5.5/5.58	OUZ99355.1	130	22(15%)	Nucleus	RNA methyltransferase activity.	0.18
188	ALBINO3-like protein 1, chloroplastic isoform X1 [*Cucumis melo*]	58/56.039	5.28/7.71	XP_008444397.1	110	23(20%)	Chloroplast	Membrane insertase activity	0.65
207	Putative 26S protease regulatory subunit 4 [*Zostera marina*]	66/68.678	4.47/9.12	KMZ68817.1	91	54(50%)	Cytoplasm	Polyubiquitin modification- dependent protein binding.	Only detected in control cell
216	ATP synthase F_O_ subunit b [*Chlorokybus atmophyticus*]	25/21.021	5.0/9.72	YP_001315093.1	105	20(62%)	Chloroplast and mitochondria	Connect F_O_, F_1_ of ATP synthesis.	Only detected in control cell
217	Ethylene-responsive transcription factor 1B-like [*Cajanus cajan*]	24/28.294	5.49/5.53	XP_020237863.1	102	17(33%)	Nucleus	DNA-binding transcription factor activity.	Only detected in control cell
225	G-box-binding factor 1 [*Arabidopsis lyrata* subsp. lyrata]	33/33.716	5.6/6.03	XP_002866995.1	86	24(22%)	Nucleus	Binds to the G-box motif (5’-CCACGTGG-3’) of the rbcS-1A gene promoter, and 5’-CACGTG-3’ of LHCB2.4 (At3g27690) promoter.	Only detected in control cell
229	DnaJ protein P58IPK homolog [*Cucurbita pepo* subsp. pepo]	40/53.895	5.65/6.85	XP_023517230.1	85	27(34%)	Endoplasmic reticulum	Chaperone (Hsp40 family). May play a role in protein folding in the endoplasmic reticulum.	Only detected in control cell
230	Tryptophanyl-tRNA synthetase [*Chlorella sorokiniana*]	39/34.287	5.69/6.97	PRW57418.1	84	23(62%)	Cytoplasm	Aminoacyl-tRNA ligase activity	Only detected in control cell
231	CCT domain-containing protein [*Cephalotus follicularis*]	48/49.843	5.77/6.17	GAV71350.1	83	29(24%)	Nucleus	Regulation of gene expression.	0.07
301	Water stress-inducible protein Rab21 [*Setaria italica*]	16/15.165	4.7/10.11	XP_004980462.1	148	17(54%)	Cytoplasm	Responsive to abscisic acid.	Only detected in bleached cell
307	Scarecrow-like protein 29 [*Camelina sativa*]	28/28.317	4.63/5.41	XP_010489817.1	82	20(27%)	Nucleus	DNA-binding transcription factor activity. Red, far-red light phototransduction	Only detected in bleached cell
310	(3S,6E)-nerolidol synthase 1 [*Herrania umbratica*]	59/59.063	5.3/6.44	XP_021294536.1	198	28(35%)	Cytosol	Stress response protein.	Only detected in bleached cell
314	G-box-binding factor 1-like isoform X3 [*Tarenaya hassleriana*]	29/33.637	5.53/6.99	XP_010548353.1	85	24(43%)	Nucleus	Regulates blue light-mediated photomorphogenic growth.	Only detected in bleached cell
319	Unnamed protein product [*Coffea canephora*]	23/27.230	6.95/5.78	CDP02738.1	92	23(34%)	N/A	N/A	Only detected in bleached cell
325	UDP-glycosyltransferase 74E2-like [*Hevea brasiliensis*]	26/25.751	6.33/4.87	XP_021651079.1	82	18(33%)	Intracellular membrane-bounded organelle	Glycosyltransferase, transferase activity, transferring hexosyl groups	Only detected in bleached cell
326	Chloroplast low molecular weight heat shock protein HSP26.2 [*Agrostis stolonifera var. palustris*]	26/26.196	6.5/7.85	AAN74534.1	118	14(47%)	Chloroplast	Stress response protein.	Only detected in bleached cell
327	Uncharacterized protein LOC104232495 [*Nicotiana sylvestris*]	27/28.821	6.7/6.15	XP_009784020.1	95	14(24%)	N/A	N/A	Only detected in bleached cell
331	Glutathione S-transferase T2-like [*Chenopodium quinoa*]	56/51.087	6.3/8.01	XP_021721044.1	120	56(54%)	N/A	Transferase activity	2.21

## Abbreviations

2DE	Two-dimensional gel electrophoresis
ABA	Abscisic aci
HSP	Heat shock protein
LC-MS/MS	Liquid chromatography–tandem mass spectrometry
LHC	Light harvesting complex
MALDI-TOF MS	Matrix assisted laser desorption ionization–time of flight mass spectrometry
ROS	Reactive oxygen species
PSII	Photosystem II
TEM	Transmission electron microscopy
